# The Association between Job Strain and Atrial Fibrillation: Results from the Swedish WOLF Study

**DOI:** 10.1155/2015/371905

**Published:** 2015-10-18

**Authors:** Eleonor I. Fransson, Magdalena Stadin, Maria Nordin, Dan Malm, Anders Knutsson, Lars Alfredsson, Peter J. M. Westerholm

**Affiliations:** ^1^School of Health Sciences, Jönköping University, 551 11 Jönköping, Sweden; ^2^Institute of Environmental Medicine, Karolinska Institutet, 171 77 Stockholm, Sweden; ^3^Department of Psychology, Umeå University, 901 87 Umeå, Sweden; ^4^Stress Research Institute, Stockholm University, 106 91 Stockholm, Sweden; ^5^Department of Internal Medicine, County Hospital Ryhov, 551 85 Jönköping, Sweden; ^6^Department of Health Sciences, Mid Sweden University, 851 70 Sundsvall, Sweden; ^7^Occupational and Environmental Medicine, Uppsala University, 751 85 Uppsala, Sweden

## Abstract

*Introduction*. Atrial fibrillation (AF) is a common heart rhythm disorder. Several life-style factors have been identified as risk factors for AF, but less is known about the impact of work-related stress. This study aims to evaluate the association between work-related stress, defined as job strain, and risk of AF. *Methods*. Data from the Swedish WOLF study was used, comprising 10,121 working men and women. Job strain was measured by the demand-control model. Information on incident AF was derived from national registers. Cox proportional hazard regression was used to estimate hazard ratios (HR) and 95% confidence intervals (CI) for the association between job strain and AF risk. *Results*. In total, 253 incident AF cases were identified during a total follow-up time of 132,387 person-years. Job strain was associated with AF risk in a time-dependent manner, with stronger association after 10.7 years of follow-up (HR 1.93, 95% CI 1.10–3.36 after 10.7 years, versus HR 1.11, 95% CI 0.67–1.83 before 10.7 years). The results pointed towards a dose-response relationship when taking accumulated exposure to job strain over time into account. *Conclusion*. This study provides support to the hypothesis that work-related stress defined as job strain is linked to an increased risk of AF.

## 1. Introduction

Cardiovascular disease (CVD) is the leading cause of death in a global perspective. According to the World Health Organization (WHO) 17.3 million people died from CVD in 2008, which represents 30% of the global deaths [1]. Atrial fibrillation (AF) is the most common cardiac arrhythmia and is also a well-confirmed risk factor of stroke [2–4]. Symptoms of atrial fibrillation include palpitations, shortness of breath, fatigue, chest pain, dizziness, and reduced physical capacity. Atrial fibrillation often affects the patients, as well as their family members, with distress and reduced well-being in their daily life [5–7]. In 2010, the estimated global age-adjusted prevalence of AF in the population of 35 years and older was 1368.5 per 100 000 in men and 856.8 in women [8]. In Europe, 3.7–4.2% of those aged 60–70 and 10–17% of those 80 years or older suffer from AF [9]. The prevalence of AF in Sweden is estimated to be 2.9% [10]. The incidence and prevalence of AF increase with age [11–14], and AF is more common in men than women [8, 11]. During the recent decades, the incidence of AF has increased, and this tendency is presumed to maintain [8].

It is not unusual that AF occurs in conjunction with other CVD (e.g., heart failure and heart valve problems) and hypertension [15]. However, AF may also occur without the impact of those factors and hereditary and life style factors are likely to play a part in the pathogenesis of AF [15–18]. Obesity, sleep apnea, heavy alcohol consumption, and prolonged physical exertion are examples of life-style factors that have been proposed as risk factors of AF [15, 18–22].

Mental stress is often reported by AF patients as a triggering factor of AF attacks [23], but few studies have evaluated the association between psychosocial stress and AF. However, in a case-control study it was found that acute life stress was related to AF risk [24], and in one recently published prospective study, an association between work-related stress and increased risk of AF was observed [25].

Although AF is common among the general population and considered as a public health disease, the knowledge about different risk factors and AF is still insufficient. The aim of this study is to provide additional knowledge about the relation between work-related stress, defined as job strain, and the onset of AF.

## 2. Material and Methods

Data were obtained from the Work, Lipids, and Fibrinogen (WOLF) study, a longitudinal occupational cohort study conducted in Sweden [26]. The original aim with the WOLF study was to investigate the associations between psychosocial work environment and cardiovascular risk factors.

### 2.1. Procedure and Participation

The baseline data collection in WOLF was carried out in the county of Stockholm during 1992–1995 (WOLF Stockholm, *n* = 5698). In 1996–1998, the data collection was extended to northern Sweden, in the counties of Västernorrland and Jämtland (WOLF Norrland, *n* = 4718). WOLF Norrland was partly established in order to recruit more blue-collar workers into the project. Altogether 36 occupational health service units in the counties of Stockholm, Västernorrland, and Jämtland were invited to participate. Of those, 33 occupational health service units accepted to participate. The occupational health service units represented approximately 60 companies in different branches comprising various occupations. However, including all employees at the 60 companies was not possible. Instead, all employees representing certain workplaces (e.g., a department, garage, institution, laboratory, and sales organization) were asked to participate. This selection was basically due to practical reasons from the perspective of the occupational health service units. Employees who were on more or less permanent leave from the workplace, for example, those stationed abroad or chronically ill, were not included in the study population. The participation rate at baseline was 82%, with higher participation rate in the northern part in Sweden.

At baseline, the participants filled in an extensive questionnaire covering different occupational aspects (e.g., work tasks, work hours, and work environment), sociodemographic aspects (e.g., education level), and lifestyle habits (e.g., smoking and physical exercise) as well as different aspects of health. In addition, a minor clinical examination was conducted by specially trained nurses at the occupational health service units. The clinical examination included measurements of height, weight, waist and hip circumference, and blood pressure. Blood samples were also collected.

A follow-up study in WOLF Norrland was conducted in 2000–2003. In total, 3633 participants from WOLF Norrland provided repeated measurements on work and life-style factors by taking part in the follow-up study.

### 2.2. Analytical Sample

In total, 10 416 working men and women participated by answering the questionnaire and taking part of the clinical examination at baseline. For the present study, we excluded participants who reported that they had experienced a myocardial infarction or heart failure prior to baseline. We also excluded those with a recorded diagnosis of AF in national hospital discharge and outpatient registers prior to baseline, leaving 10 121 participants (6971 men and 3150 women) as our analytical sample. In the analyses with repeated measurements, 3123 participants were included.

### 2.3. Work-Related Stress according to the Demand-Control Model

Work-related stress was defined according to Karsek's job demand-control model [27, 28], which is characterized by the combination of psychological job demands and control over the work situation. The model proposes that those experiencing high job demands in combination with low control (i.e., high strain or job strain) are in a stressful work situation and are at higher risk for developing ill health. In this study we used the Swedish demand-control questionnaire containing five job demands items and six control items to measure job strain [29]. Cronbach's alpha for the job demands and job control subscales was 0.72 and 0.74, respectively. Mean response scores for the job demands and for the job control items were computed for each participant. We used the median scores as cut-points for high and low demands (“high demands” being defined as scores strictly above the study-specific median score) and job control (“low control” being defined as scores strictly below the study-specific median score). In the analyses we used both a dichotomized measure of job strain (high strain versus all others) and four categories based on the combination of job demands and control: low strain jobs (low demands, high control); passive jobs (low demands, low control); active jobs (high demands, high control); and high strain jobs (high demands, low control).

### 2.4. Atrial Fibrillation

Information on incident AF, or flutter, was derived from the Swedish national hospital discharge, outpatient, and mortality registers by using the following ICD codes: ICD-10 code I48; ICD-9 code 427D; and ICD-8 code 427.92.

### 2.5. Potential Confounding and Mediating Factors

In addition to age in years (continuous), sex, and part of study (Stockholm, Norrland), we considered the following factors as potential confounding or mediating factors: socioeconomic status (manual workers, lower level/intermediate nonmanual employees, and professionals), exercise (seldom, sometimes, and regularly), smoking (never (neither current nor ex-smoker), ex-smoker (has previously smoked for at least one year but is not a current smoker), and current smoker), alcohol consumption (none (0 units/week), moderate (1–14 units/week for women, 1–21 units/week for men), intermediate (15–20 units/week for women, 22–27 units/week for men), and heavy (21 units/week or more for women, 28 units/week or more for men)), waist circumference (<94 cm (men) or <80 cm (women), 94–101.99 cm (men) or 80–88 (women), and >102 cm (men) or >88 cm (women)), and hypertension (systolic blood pressure ≥140 mmHg, or diastolic blood pressure ≥90 mmHg, or self-reported treatment with antihypertensive drugs).

### 2.6. Statistical Analyses

The participants were followed up from their baseline assessment of job strain to the first registered AF event, migration out of Sweden, death, or end of follow-up, whichever came first. Independent *t*-tests and Chi^2^-tests for bivariate analyses were conducted in order to test potential differences in baseline characteristics between participants with and without AF. Cox proportional hazard regression was used to estimate hazard ratios (HR) and 95% confidence intervals (CI) to quantify the relationship between job strain and risk of atrial fibrillation. All analyses were adjusted for age, sex, and part of study. Other potential confounding and mediating factors were added one by one to the age, sex, and part of study adjusted Cox proportional hazard regression model. Only those factors changing estimates of job strain versus others with more than 10% were to be included in subsequent models [30]. To evaluate a potential effect modification by sex, a stratified analysis by sex was carried out, as well as including a statistical interaction term between job strain and sex in the Cox proportional hazard regression model. Kaplan-Meier curves were used to inspect the proportionality of hazards over time. Analyses of accumulated exposure to job strain and the risk of AF were carried out in a subsample of the WOLF Norrland study population for whom repeated measures of job strain were available. In the analyses with repeated measures, the start of follow-up time was set at the date of the second data collection. A *P* value for trend was derived by including the variable on accumulated job strain as a continuous variable with three levels in the Cox proportional hazard regression model. All data analyses were carried out using SAS version 9.2.

### 2.7. Ethics

All participants gave informed consent to participate in the study. The WOLF study has been approved by the Ethics Committee at Karolinska Institutet, Stockholm (# 92-198), and the Regional Ethical Review Board in Stockholm (# 2006/257-31, # 2008/1638-31/5).

## 3. Results

Characteristics of the study sample are presented in Table 1. During a total follow-up time of 132,387 person-years (median follow-up time 13.6 years), 253 incident AF events were recorded. Compared to participants without AF, participants with AF were more likely to be male, older, and current or ex-smokers and have higher waist circumference and more likely to suffer from hypertension.

In the age, sex, and part of study adjusted Cox proportional hazard regression model, job strain was associated with a 38% increased risk of AF when compared to all others, although the association was not statistically significant (HR 1.38, 95% CI 0.95–2.00) (Table 2). None of the investigated potential confounding or mediating factors changed the estimated HR with more than 10% and were therefore not included in the regression model. No clear effect modification by sex was observed (HR 1.42, 95% CI 0.94–2.14 for men, HR 1.22, 95% CI 0.51–2.92 for women, *P* value for interaction = 0.75). When using the four demand-control categories, high strain was associated with a 50% increased risk of AF compared with the low strain group, the result being borderline significant (HR 1.50, 95% CI 0.99–2.27) (Table 2).

**Figure 1:**
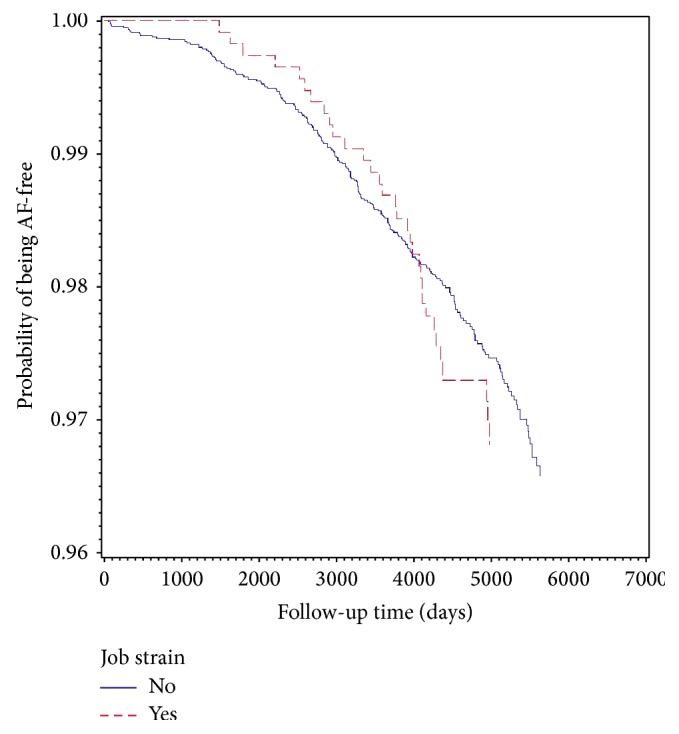
Unadjusted Kaplan-Meier plot, job strain versus no strain.

When inspecting the crude Kaplan-Meier plot, it was observed that those with job strain had a slightly better probability of being AF-free during the first years of follow-up as compared with the nonstrain group, but the curves crossed at approximately 10.7 years after baseline (Figure 1). This led us to do stratified analysis, splitting the follow-up period at 10.7 years after baseline. The seemingly lower AF risk in the job strain group during the first follow-up period was mainly explained by higher prevalence of job strain among women than men (14% versus 10%) and that the job strain group tended to be younger than the nonstrain group (mean age 41.2 versus 42.7 years). After adjusting for age, sex, and study part, the HR for job strain versus others was 1.11 (95% CI 0.67–1.83) during the first part of the follow-up period. In the analysis based on the follow-up period after 10.7 years, it was observed that job strain versus all others was significantly associated with the risk of AF in the age, sex, and study part adjusted models (HR 1.93 95% CI 1.10–3.36) (Table 2). The same pattern was seen when using the four demand-control categories, where the HR for the high strain group compared with low strain was 2.13 (95% CI 1.13–4.04) (Table 2).

For a subsample of the WOLF Norrland study population we had repeated measurements of job strain, measured at baseline (1996–1998) and follow-up (2000–2003). Taking into account the exposure to job strain at none (*n* = 2472, AF cases = 34), one (*n* = 527, AF cases = 10), or both measurement occasions (*n* = 124, AF cases = 3), we observed an association between job strain and AF risk in a dose-response manner (Table 3). However, as the number of participants and AF cases exposed at both occasions was few, the estimates were imprecise.

## 4. Discussion

In this study, we observed an association between work-related stress, defined as job strain, and the risk of atrial fibrillation. The association was time-dependent and more pronounced at the end of the follow-up period. The risk of AF was approximately two times higher among those exposed to job strain compared to those unexposed during the latter part of the follow-up period. In analyses taking repeated measurements into account, our results were suggestive of a dose-response relationship between accumulated exposure to job strain and AF risk.

Published studies on the association between psychosocial stress in general and work-related stress in particular and AF are scarce. We are only aware of one previously published study on job strain and AF risk [25]. That recently published study by Torén et al. was also based on a Swedish sample but only included men and used a job exposure matrix based on occupation at baseline to measure job strain. They found a 32% increased risk associated with being exposed to job strain versus all others (HR 1.32, 95% CI 1.003–1.75) [25], which is in accordance with our overall HR estimate of 1.38. In another study, mental stress in terms of acute life stress was found to be related to AF risk [24], and in a study conducted among AF patients, mental stress was the most frequent reported triggering factor of AF attacks [23]. Some case reports linking emotional stress to AF have also been published [31, 32]. In addition, different aspects of work-related stress, including job strain, effort-reward imbalance, and job insecurity, have been linked to increased risk of coronary heart disease [33–35].

The biological pathway between work-related stress and AF is not clear. Ectopic foci in pulmonary veins are recognized as triggers of AF, and the processes leading to the onset of AF include atrial fibrosis, structural remodeling of the heart tissue, and inflammation [36]. Altered sympathetic and parasympathetic balance and neurohormonal activation have also been proposed to play key roles in the development of AF [36, 37]. In a study on 77 AF patients, Bettoni and Zimmerman showed an increase in adrenergic tone followed by a marked shift towards vagal predominance immediately before the onset of paroxysmal AF [38], and Patterson et al. showed in an experimental study on dogs that both the parasympathetic and sympathetic nervous system have a role in initiating and triggering pulmonary vein activity [39]. Reactions to stress include several physiological responses involving both the hypothalamic-pituitary-adrenal axis and the autonomous nervous system [40, 41]. Responses include increased release of glucocorticoid hormones, such as cortisol, and increased sympathetic activity, with increased release of adrenaline and noradrenaline. An effect on inflammation has also been observed [40]. These factors are making a link between psychosocial stress, including work-related stress, and AF plausible. Atrial fibrosis and structural remodeling develop over time and may be asymptomatic for several years. This may explain our finding with a stronger association between job strain and AF observed at the later part of the follow-up period and that accumulated exposure to job strain over time seems to be associated with higher risk as compared to shorter episodes of exposure, although the exact mechanism behind this observation is not clear.

Our study has several strengths, including the prospective design, being based on a large sample of working people, and including both men and women. A high participation rate and low internal dropout are further strengths. We used a well-established measure on work-related stress, based on the demand-control or job strain model, frequently used in studies on work-related stress and health related outcomes. Job strain was measured by self-report through a validated questionnaire [29, 42]. The outcome was defined through national registers with high quality and coverage [43]. One major advantage with our study is that we had access to repeated measures of job strain for a subset of our study sample. We also had access to several potential confounding factors, which we could take into account in the analyses. Indeed, participants diagnosed with AF during the follow-up period were to a higher degree male, older, smoker, and obese and were more likely to suffer from hypertension, which is in accordance with previous studies [8, 11, 15, 18]. However, after adjusting for age, sex, and study part, taking into account life-style factors, obesity or hypertension did not change the estimated association in any substantial way. However, it is important to acknowledge that an unfavorable work situation may affect life-style factors in a longitudinal perspective [44], potentially contributing to the association between long-term exposure to job strain and ill health.

Our study also has some limitations. Despite the large study sample and a rather long follow-up period (median follow-up time: 13.6 years), the number of AF cases was relatively low, limiting the power of the study. This is especially true for the longitudinal analyses taking repeated measures into account. Also, the majority of the incident AF cases in our study had an unspecified AF diagnosis, preventing more detailed analyses of AF subtypes such as paroxysmal, persistent, and chronic AF. Furthermore, the experience and perception of stress at work is a complex issue, and there are several ways of operationalizing work-related stress. Here, we used the most frequently utilized model, the job strain model. However, there are several other models and operationalizations available covering other aspects of work-related stress, such as the effort-reward imbalance, job insecurity, and organisational injustice, which is not covered in the present study. Evaluating other aspects of work-related stress in relation to AF in addition to the job strain model will yield a more complete picture of the association between work-related stress and AF.

## 5. Conclusion

Our study lends some support to the hypothesis that work-related stress, defined as job strain, is related to the development of AF over time. Our results suggest that the association may be time-dependent and that long-term exposure to job strain may be more strongly associated with AF risk than shorter bouts of exposure.

## Figures and Tables

**Table 1:** Baseline characteristics in the total study sample and among participants with and without atrial fibrillation (AF), the WOLF study, Sweden.

Characteristics	Total *n* = 10121	Not AF *n* = 9868	AF *n* = 253	*P* value^*^
Age, mean (sd)	42.5 (10.7)	42.3 (10.7)	51.3 (8.3)	<0.001
Sex, *n* (%)				
Men	6971 (69)	6757 (68)	214 (85)	<0.001
Women	3150 (31)	3111 (32)	39 (15)	
Study part, *n* (%)				
Stockholm	5518 (55)	5368 (54)	150 (59)	0.12
Norrland	4603 (45)	4500 (46)	103 (41)	
Demand-control, *n* (%)				
No strain	8960 (89)	8739 (89)	221 (87)	0.55
Job strain	1161 (11)	1129 (11)	32 (13)	
Low strain	3192 (32)	3112 (32)	80 (32)	0.64
Passive	3259 (32)	3186 (32)	73 (29)	
Active	2509 (25)	2441 (25)	68 (27)	
High strain	1161 (11)	1129 (11)	32 (13)	
SES, *n* (%)				
Manual workers	4422 (44)	4310 (44)	112 (44)	
Lower level/ intermediate nonmanual employees	4341 (43)	4242 (44)	99 (39)	0.09
Professionals	1234 (12)	1192 (12)	42 (17)	
Physical exercise, *n* (%)				
Seldom	2488 (25)	2420 (25)	68 (27)	0.64
Sometimes	3876 (38)	3781 (38)	95 (38)	
Regularly	3727 (37)	3639 (37)	88 (35)	
Smoking, *n* (%)				
Never smokers	4739 (48)	4664 (48)	75 (30)	<0.001
Ex-smokers	2893 (29)	2788 (29)	105 (42)	
Current smokers	2250 (23)	2182 (23)	68 (27)	
Alcohol consumption, *n* (%)				
Non	487 (5)	470 (5)	17 (7)	0.37
Moderate	8688 (88)	8476 (88)	212 (87)	
Intermediate	318 (3)	311 (3)	7 (3)	
Heavy	401 (4)	394 (4)	7 (3)	
Waist circumference				
≤94.0 (M); ≤80.0 (W)	6322 (63)	6211 (63)	111 (44)	<0.001
94–101.99 (M); 80–88 (W)	2241 (22)	2174 (22)	67 (26)	
>102 (M); >88 (W)	1511 (15)	1436 (15)	75 (30)	
Hypertension, *n* (%)				
No	8043 (80)	7891 (80)	152 (60)	<0.001
Yes	2062 (20)	1961 (20)	101 (40)	

^∗^Chi^2^-tests for comparison of proportions, *t*-test for comparisons of continuous variable.

**Table 2:** The estimated association between job strain and the risk of atrial fibrillation. Hazard ratios (HR) with 95% confidence intervals (95% CI).

Work-related stress	HR (95% CI)^*^	HR (95% CI)^*^	HR (95% CI)^*^
	Complete follow-up, 253 events	First 10.7 years of follow-up, 165 events	Follow-up after 10.7 years, 88 events
No strain	1 (ref)	1 (ref)	1 (ref)
Job strain	1.38 (0.95–2.00)	1.11 (0.67–1.83)	1.93 (1.10–3.36)

Low strain	1 (ref)	1 (ref)	1 (ref)
Passive	1.08 (0.79–1.49)	1.05 (0.71–1.54)	1.16 (0.66–2.03)
Active	1.21 (0.87–1.67)	1.22 (0.82–1.83)	1.20 (0.67–2.09)
High strain	1.50 (0.99–2.27)	1.19 (0.69–2.06)	2.13 (1.13–4.04)

^∗^Adjusted for age, sex, and part of study.

**Table 3:** The estimated association between accumulated exposure to job strain and the risk of atrial fibrillation. Hazard ratios (HR) with 95% confidence intervals (95% CI), based on a subsample from the WOLF Norrland study population with baseline measure in 1996–1998 (t1) and repeated measure in 2000–2003 (t2), *n* = 3123.

	HR (95% CI)^*^	*P* value for trend
Subsample with repeated measurements, 47 events		
Unexposed to job strain at both t1 and t2	1 (ref)	0.06
Job strain at either t1 or t2	1.68 (0.83–3.40)	
Job strain at both t1 and t2	2.28 (0.70–7.44)	

^∗^Adjusted for age and sex.
